# Supplementary material for: Mechanism of RNA polymerase III termination-associated reinitiation-recycling conferred by the essential function of the N terminal-and-linker domain of the C11 subunit
Source: Nat Commun. 2021 Oct 8;12:5900. doi: 10.1038/s41467-021-26080-7 (PMC8501072; doi:10.1038/s41467-021-26080-7)
Supplement: Supplementary file 1 — Supplementary Information [file 41467_2021_26080_MOESM1_ESM.pdf]

Supplementary Information for

Mechanism of RNA Polymerase III termination-associated reinitiation-recycling conferred by the essential function of the N terminal-and-Linker domain of the C11 subunit

Authors: Saurabh Mishra[1], Shaina H. Hasan[2], Rima M. Sakhawala[3], Shereen Chaudhry[4] and Richard J. Maraia*

Affiliation: Intramural Research Program of the *Eunice Kennedy Shriver* National Institute of Child Health and Human Development, National Institutes of Health, Bethesda, MD USA,

*Corresponding author E-mail: maraiar@mail.nih.gov

[1]Current address: Department of Biochemistry, Banaras Hindu University, Varanasi, India. [2]Mayo Clinic Alix School of Medicine, Scottsdale, Arizona, [3]Section on Regulatory RNA, National Institute of Diabetes and Digestive and Kidney Diseases, Bethesda, MD. [4]Pfizer (Pearl River Site), 401 N Middletown Rd, Pearl River, NY

This PDF contains Supplementary Figures 1-7, Supplementary Table 1, and References to literature cited.

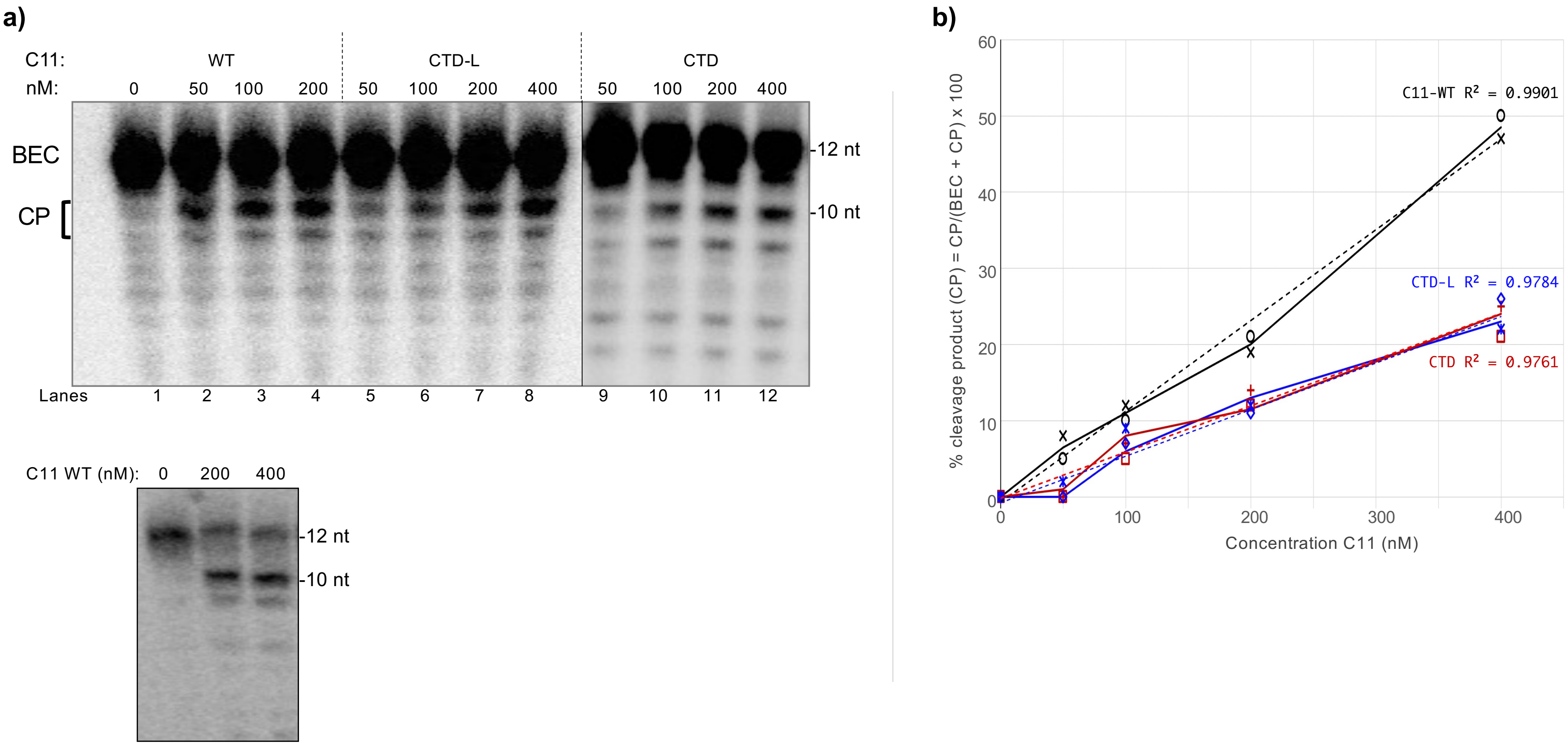

Supplementary Figure 1. Concentration-dependent RNA 3' cleavage by the isolated CTD of C11. Related to Fig. 2d and e. **a)** BECs (backtracked elongation complexes) were incubated with varying concentrations (in nM) of C11, CTD-L or CTD as indicated above the lanes, for 15 min prior to addition of $MgCl_2$, then further incubated and processed as described in figure 2c-d; cleavage products (CP) are indicated by brackets. Positions of 12 nt and 10 nt RNA markers are shown. The lower image shows a gel for a cleavage assay performed with higher concentrations of C11. **b)** The quantified data in main figure 2e are represented here by the plotted points and after being subjected to linear regression analysis using Excel; the calculated R^2 values for the fitted dashed trendlines are shown. N = 2 for each concentration point, representative of biological replicates, as in main figure 2e.

Supplementary Fig. 2, Mishra et al.

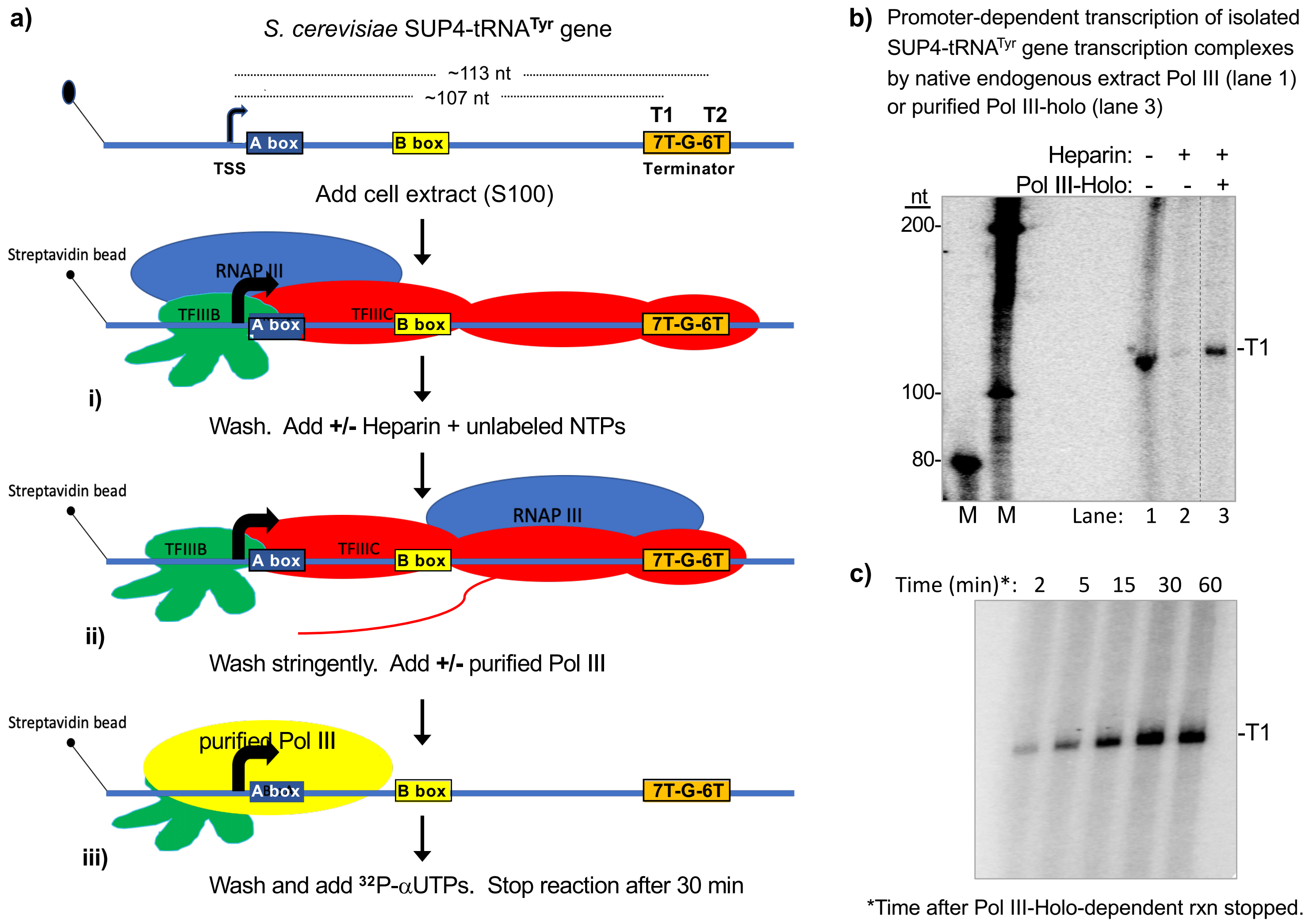

Supplementary Figure 2. Isolation of stable transcription complexes and assay for Pol III recycling. Related to Fig. 4. Proof of principle for promoter dependent transcription reinitiation-recycling. **a)** Illustration describing preparation of Pol III-depleted stable transcription initiation complexes. A 5' biotinylated SUP4-tRNA^{Tyr} gene-containing DNA fragment was incubated with *S. cerevisiae* cell extract (S100) to allow formation of stable preinitiation complexes. This was followed by three wash-incubation-wash steps; **i)** The streptavidin-immobilized complexes were washed and incubated with 500 μM of all four NTPs (each NTP non-radioactive) +/- 0.03 mg/ml heparin to strip off, capture and **ii)** wash away the native Pol III. Next, +/- purified Pol III-holo was added in excess, incubated for 20 min and the excess was **iii)** washed away before NTPs containing ^{32}P-αUTP was added. Note that the promoter-dependent T1 and T2 transcription products would be approximately 107 and 113 nucleotides (nt) in length. **b)** Lane 1 shows products of residual extract-derived Pol III (no heparin was included in wash **i** for the purposes of this proof of principle experiment only); lane 2 shows products after extract-derived Pol III was stripped away (with heparin in wash **i**), and lane 3 shows products dependent on addition of purified Pol III-holo, as indicated above the lanes. Lane 3 was from same experiment, same gel but juxtaposed to lane 2 as indicated by the vertical line. Lanes M contain ^{32}P 5' end labeled single-stranded oligo-DNA size markers as indicated to the left in nucleotides. These markers were also used in gels with products of other transcription reactions that produced T2 band (e.g. main figures 4b, c, 5a, c, 6a) as illustrated in panel a above. **c)** Products of a reinitiation-recycling assay dependent on Pol III-holo transcription of the SUP4-tRNA^{Tyr} transcription complexes showing different time points taken from a reaction as in panel B, lane 3, described in the text.

Supplementary Fig. 3, Mishra et al.

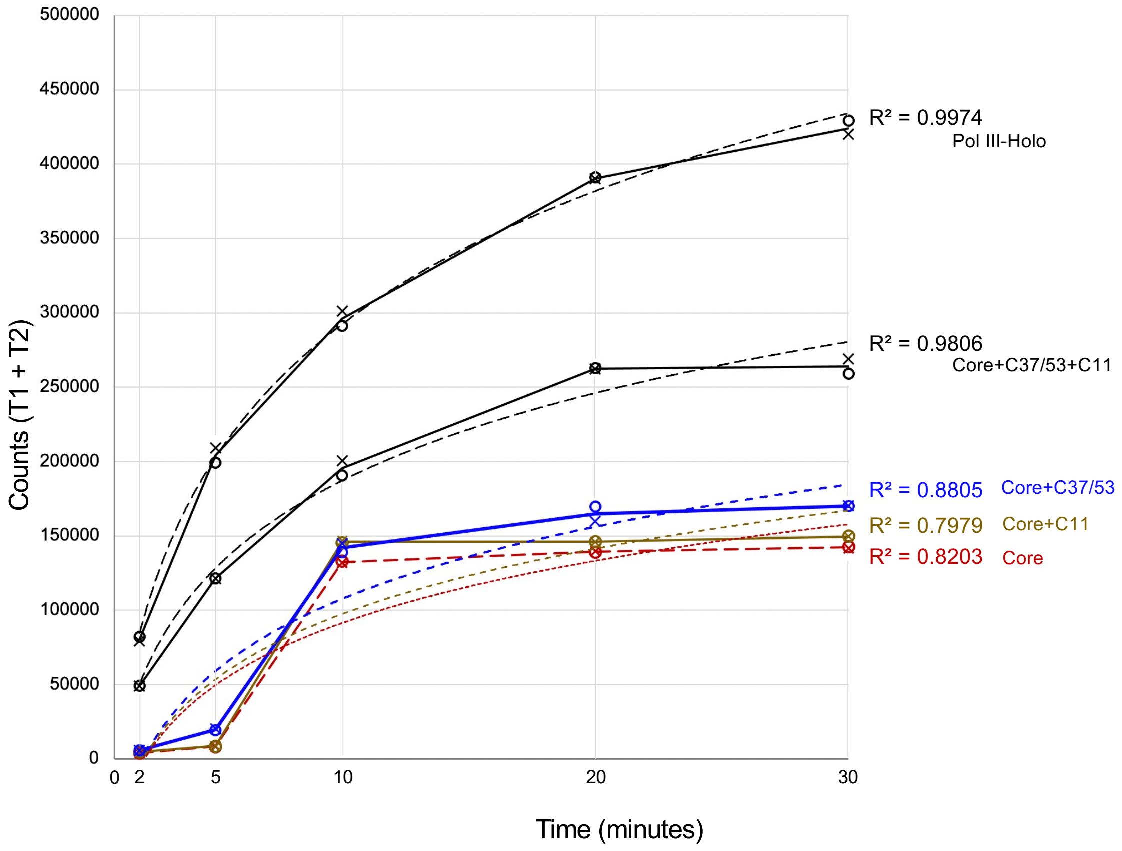

Supplementary Figure 3. Pol III reinitiation-recycling dependency on C37/53 and C11. Related to Fig. 4c. Amounts of T1+T2 RNAs at each time point in Fig. 4b represented here by the plotted points, were subjected to regression analysis using Excel with the calculated R^2 values for the logarithmic fitted trendlines (dashed) shown. N = 2 for each point, representative of biological replicates. See figure 4c for other details.

Supplementary Fig. 4, Mishra et al.

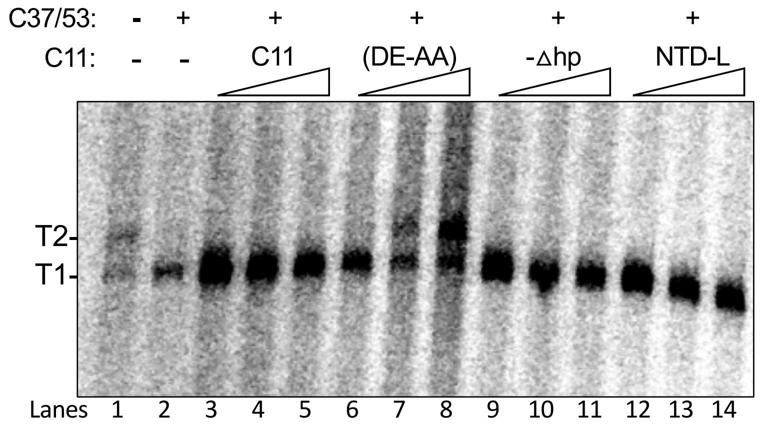

Supplementary Figure 4. Concentration-dependent Pol III termination inhibition by C11 CTD hairpin mutant D91A E92A (DE-AA) but not other cleavage-deficient mutants. Related to Figs. 5a and 6a. Products of *in vitro* transcription reactions using the SUP4-tRNA^{Tyr} complexes and Pol III-core alone (lane 1), or Pol III-core preincubated for 20 min with C37/53 and increasing concentrations of C11 or its derivatives (100, 200 and 500 nM) as indicated above the lanes. The T1 and T2 bands are as described for figure 4b and d.

Supplementary Fig. 5, Mishra et al.,

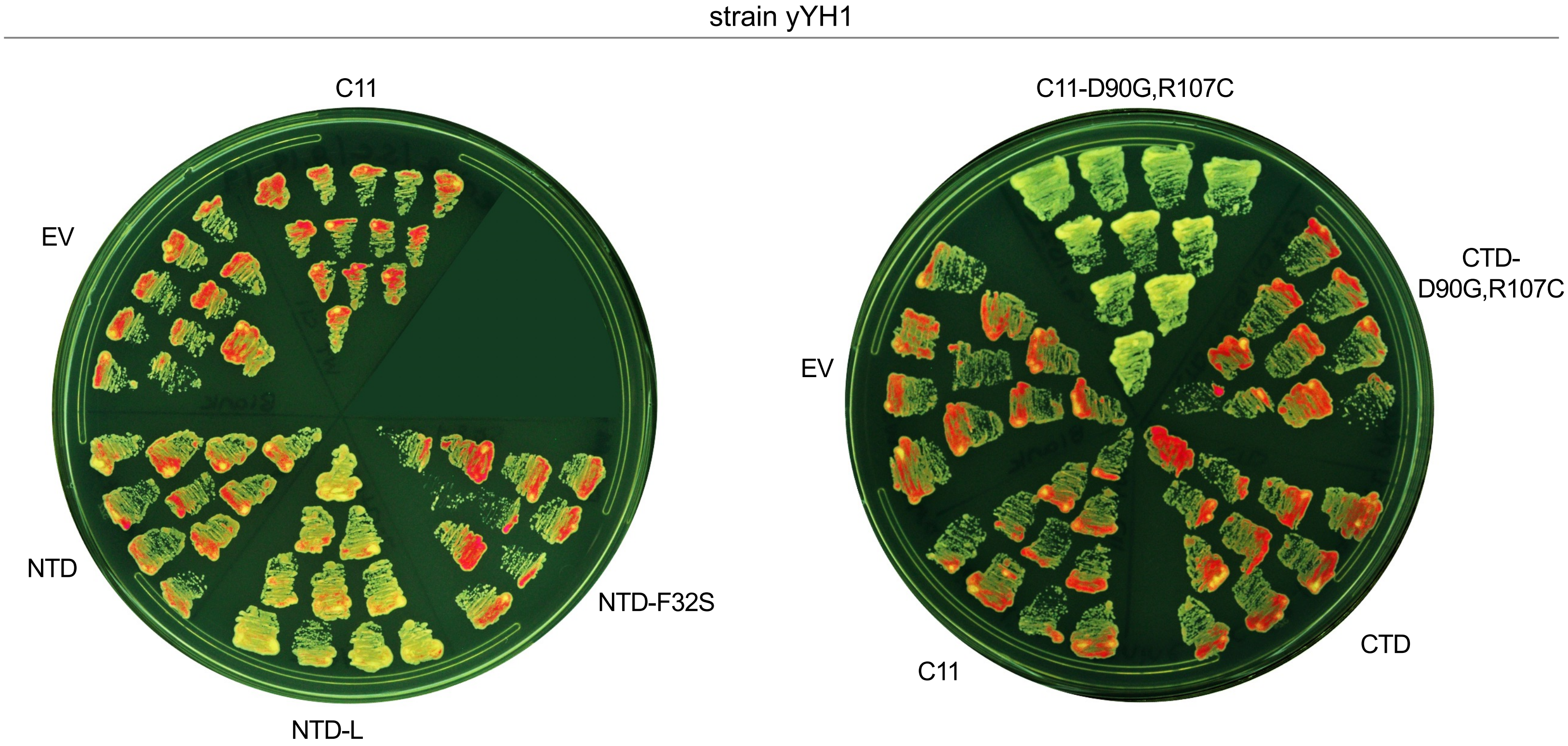

Supplementary Figure 5. Evidence that NTD-L exhibits transcription initiation-positive cleavage-deficient activity *in vivo.* Related to Fig. 8. Red-white tRNA-mediated suppression in *S. pombe* yYH1 using pRep4X-mediated expression of C11, its different derivatives or the empty vector (EV) in yYH1. After initial transformation, multiple colonies were streaked onto EMM-complete lacking uracil with limiting adenine (10 mg/L) and grown at 32°C for color development. This strain contains a suppressor-tRNA allele with a 7T terminator whose level of suppression (light colonies) reflects Pol III-initiated transcription whose termination occurs despite deficiency of C11-mediated RNA 3'-cleavage activity[1] (see text).

Supplementary Fig. 6, Mishra et al.,

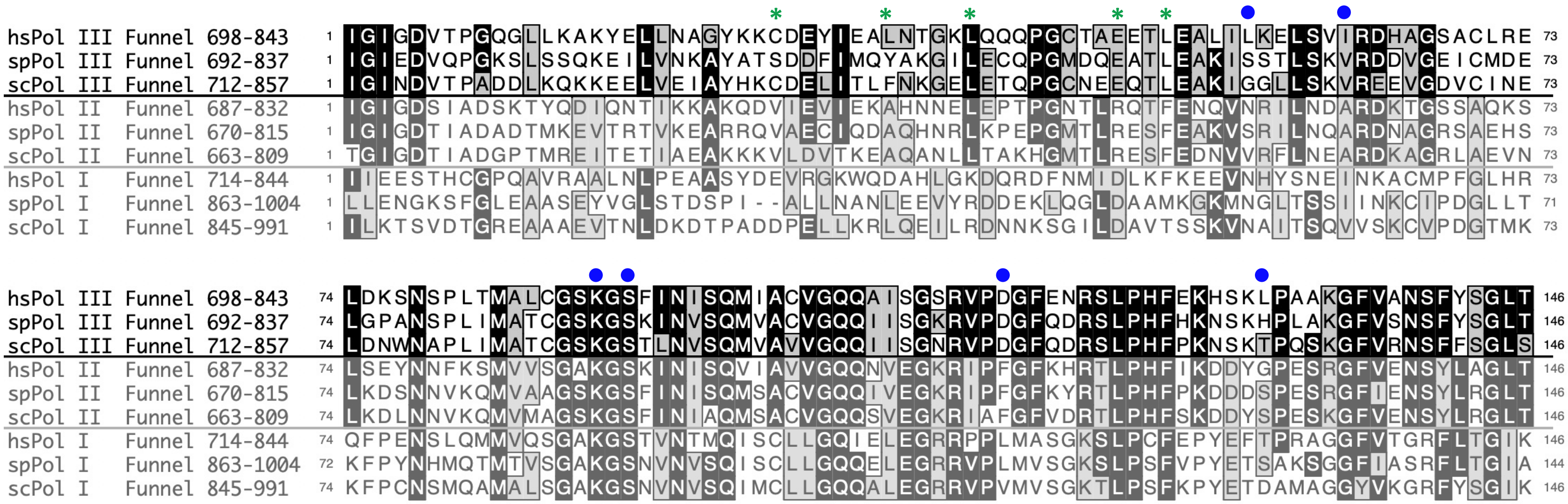

Supplementary Figure 6. Conservation of funnel residues involved in yeast Pol III termination-recycling in close proximity to the human RPC10 distal linker. Multiple sequence alignment of the funnel helices of RPC1 (Pol III), RPB1 (Pol II) and RPA1 (Pol I), with the mutations found in *S. pombe* Pol III termination *rpc1*-mutants[2] that are highlighted in the cryo-EM structures in Supp Figures S7A-F. The single point mutations found in mutants associated with loss-of function (LOF) i.e., readthrough of a 5T terminator are indicated by green asterisks above the sequences, and mutations associated with gain-of-function (GOF) at a 4T terminator and slow growth are indicated by blue circles above the sequences.. This alignment is shown here as an extended version of that previously available[2]. In reference [2], a limited number of slow growth mutants were obtained of which two were in the invariable catalytic Asp-triad motifs, and more in the funnel helices (blue circles in alignment and blue spheres in Supp Fig. 7e and f) and the rest in the trigger loop itself; we consider this class as having negative effects on pol III enzymatic function. The other class of mutants were much more numerous and were fast growing; characterization of the prototypical of which revealed higher levels of certain Pol III transcripts than the wild-type cells[2].

Supplementary Fig. 7, Mishra et al.

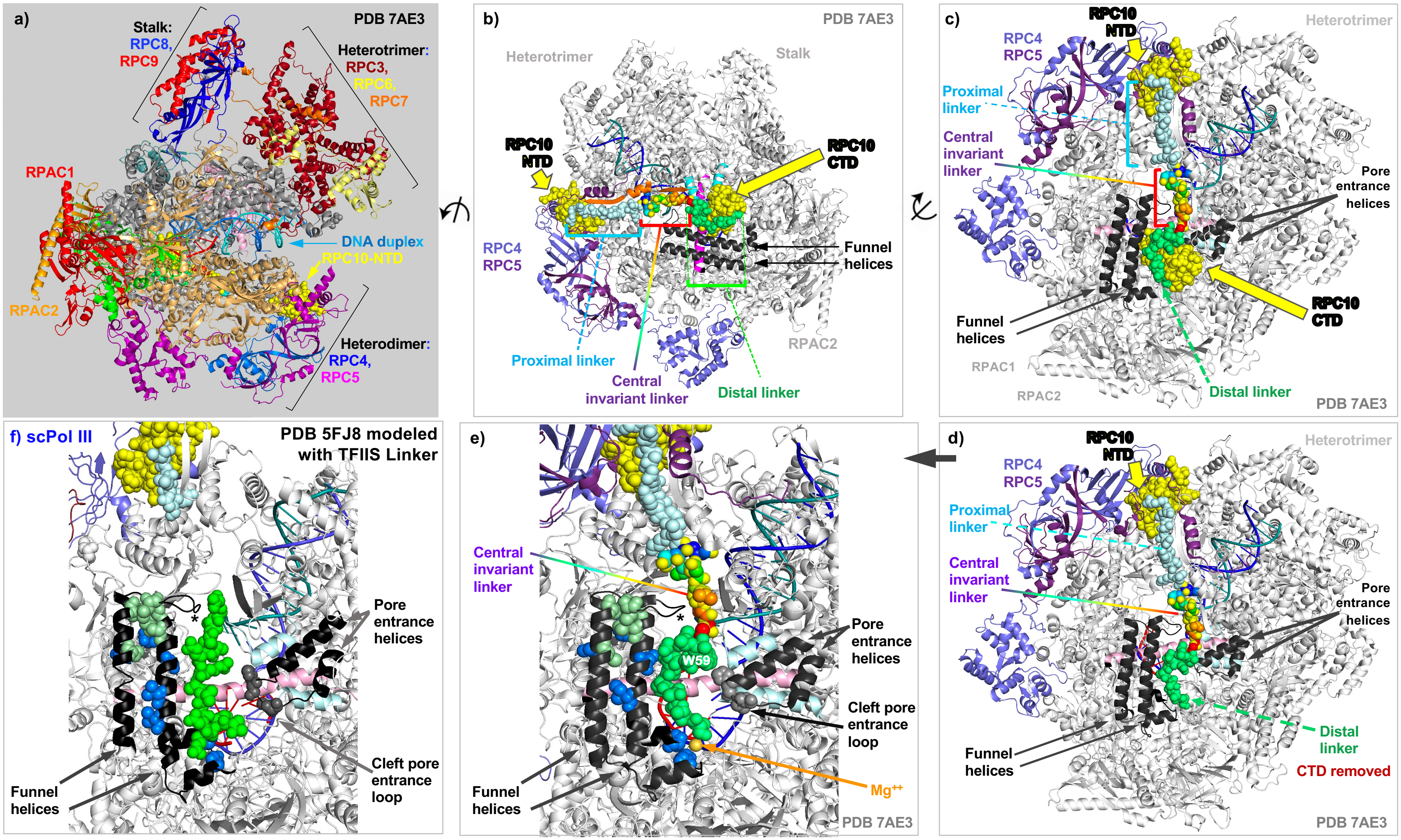

Supplementary Figure 7. Structural conservation of funnel and pore entrance residues involved in Pol III termination and recycling in proximity to the human RPC10 distal linker. Cryo-EM structures of the human Pol III elongation complex in which part of the Linker and CTD of RPC10 (C11 homolog) were described as inside the funnel (PDB 7AE3)[3] as relevant to this study. **a)** The orientation and coloring here is a standard view, as in figure 1c in reference [3]. The RPC10 subunit is shown as spheres, which in this view mostly reveals the NTD, in close proximity to its associated RPC5/RPC4 heterodimer, as annotated. The DNA duplex is also annotated. **b)** A rotated view reveals RPC10 (as spheres) with its extended linker, colored as follows: the NTD and CTD are yellow, the three regions of the linker as detailed in figure 1 are annotated and indicated by brackets; the 11 amino acids (aa) of the proximal linker are pale cyan, the 9 aa of the central invariant linker are multicolor rainbow, and the 13 aa of the distal linker are bright green. The globular Cys-4 Zn region of the CTD is apparent as it blocks view of the hairpin pointing toward the active center in front of it. A kink about half way along the distal linker comes in close contact of the two funnel helices, colored black. The bridge helix (BH) is colored magenta, and the two trigger loop (TL) helices cyan. **c, d)** Rotated views that differ from each other in that the entire CTD of RPC10 has been removed from the latter to better reveal the extent of the distal linker (green) inside the funnel at the pore entrance in preparation for panel e in which previously mapped mutations in *S. pombe rpc1-termination* mutants[2] are highlighted (see Supp Fig 6). The bridge helix (BH) and trigger loop (TL) helices are light pink and pale cyan respectively. The pore entrance helices are colored black. **e)** Enlarged view of panel d, also from PDB 7AE3 human Pol III EC, in which mutations found in the funnel helix region of *S. pombe rpc1-termination* mutants (see Supp Fig. S6) are highlighted by representation as colored spheres. The BH traversing horizontally behind the distal linker and funnel helices is light pink and the TL helices are pale cyan. Two termination-disrupting mutations found at the cleft pore entrance loop (CPEL, H1346, M1348)[2] are indicated as gray spheres connecting the pore entrance helices. Termination-disrupting mutations in the funnel helices and associated funnel elements are highlighted as blue and green[2] spheres (Supp Fig. S6). A loop connecting the top of the funnel helices is indicated by an asterisk which comprises one part of a narrow grove through which the central linker passes, a down pointing beta-strand (grey) is on the other side [4]. **f)** Similar view as panel e but of the *S. cerevisiae* Pol III EC containing the scC11 NTD (yellow) and part of proximal linker (pale cyan) (PDB 5FJ8)[5], into which the distal linker-CTD domain of TFIIS had been modeled, [see figures 3C and D in ref 2]; for panel f here, the CTD was removed for comparison to panel e.

Supplementary Table 1. Oligo DNAs and RNAs used in this study.

In vitro Transcription					
Name	Sequence (5' to 3')	Terminator	Strand	Figs.	Remarks
AGTO7X	AACAATTAAATACTCTCTATTATCCATAGGCGGACGACGAAAGGCGCAAGACTT TTTTTTTGTCTCCAAGACTTGCTCGC	9T	NT	Figs 2, 5	Cleavage Assay
AGTO16X	GCGAGCAAGTCTTGGAGACAAAAAAAAAGTCTTGCGCCTTTCGTCGTCCGCCTA TGGATAATAGAGAGTATTTAATTGTT	9T	T	Figs 2, 5	Cleavage Assay
SMO27X	AACAATTAAATACTCTCTATTATCCATAGGCGGACGACGAAAGGCGCAAGACTT TTTAGAAGTCTCCAAGACTTGCTCGC	5T	NT	Fig. 3	Term assay
SMO77	GCGAGCAAGTCTTGGAGACTTCTAAAAAGTCTTGCGCCTTTCGTCGTCCGCCTA TGGATAATAGAGAGTATTTAATTGTT	5A	T	Fig. 3	Term Assay
RNA6	CGGACGACGAUU	RNA primer		Figs 2, 5	Cleavage
RNA1	CGGACGACGA	RNA primer		Fig. 3	Term assay
Cloning Oligos					
SMO252	ATGTAggatccGCTTTCGTTTTGTCCTTCGTG	Forward Primer with BamHI for scC11			
SMO253	CGATCTCGAGTTAATTTTCCTTCCATCTATGAC	Reverse primer with XhoI for scC11			
SMO254	CGATCTCGAGTTAGTCCCAACCACC ACCAAGAAC	Reverse primer with XhoI for scNTDL C11			
SMO255	ATGTAggatccGGGTATAGAAATCTATGATAGG	Forward Primer with BamHI for scCTDL C11			
SMO256	ATGTAggatccTGTGGATCAAACCAAAACCCAATGTCCC	Forward Primer with BamHI for scCTD C11			
SMO261	AAAGGAAGTTgccgccGTTCTTGGTGGTG	Forward primer for SDM ScC11 D52A, D33A			
SMO262	CTTGGAAGTTTCTTCCTATC	Reverse primer for SDM ScC11 D52A, D33A			
SMO243	TAGGGATCCCCGGGTAAAG	Forward primer for deletion of CTD of spC11			
SMO244	AAAAGCTTCTTCTCCACCTAATAC	Reverse Primer for deletion of CTD of spC11			
SMO245	CTCTACAGTCGTCACGAATTTG	Forward primer for deletion of NTD of spC11			
SMO246	CATCTCGAGGTATGATTTAACAAAG	Reverse Primer for deletion of NTD of spC11			

References for citations in supplemenrtary figures.

1. Huang, Y., Intine, R.V., Mozlin, A., Hasson, S. & Maraia, R.J. *Mol Cell Biol* **25**, 621-36. (2005).

2. Rijal, K. & Maraia, R.J. *PLoS Genet* **12**, e1006253 (2016).

3. Girbig, M. et al. *Nat Struct Mol Biol* **28**, 210-219 (2021).

4. Wang, Q. et al. *Nat Struct Mol Biol* **28**, 220-227 (2021).

5. Hoffmann, N. et al. *Nature* **528**, 231-6 (2015).
