# Supplementary material for: Mechanism of RNA polymerase III termination-associated reinitiation-recycling conferred by the essential function of the N terminal-and-linker domain of the C11 subunit
Source: Nat Commun. 2021 Oct 8;12:5900. doi: 10.1038/s41467-021-26080-7 (PMC8501072; doi:10.1038/s41467-021-26080-7)
Supplement: Supplementary file 3 — Source Data [file 41467_2021_26080_MOESM3_ESM.pdf]

Mishra et al., and Maraia, Pol III – C11, *Nature Communications*. Raw Images file

Uncropped gel for Fig 2B

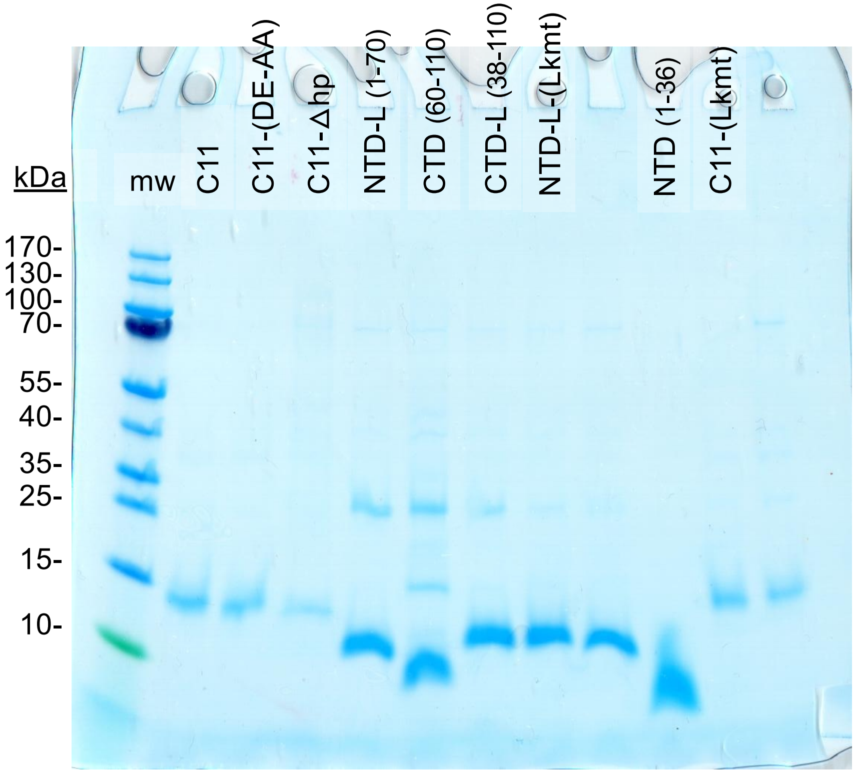

Uncropped gels for Fig 2D

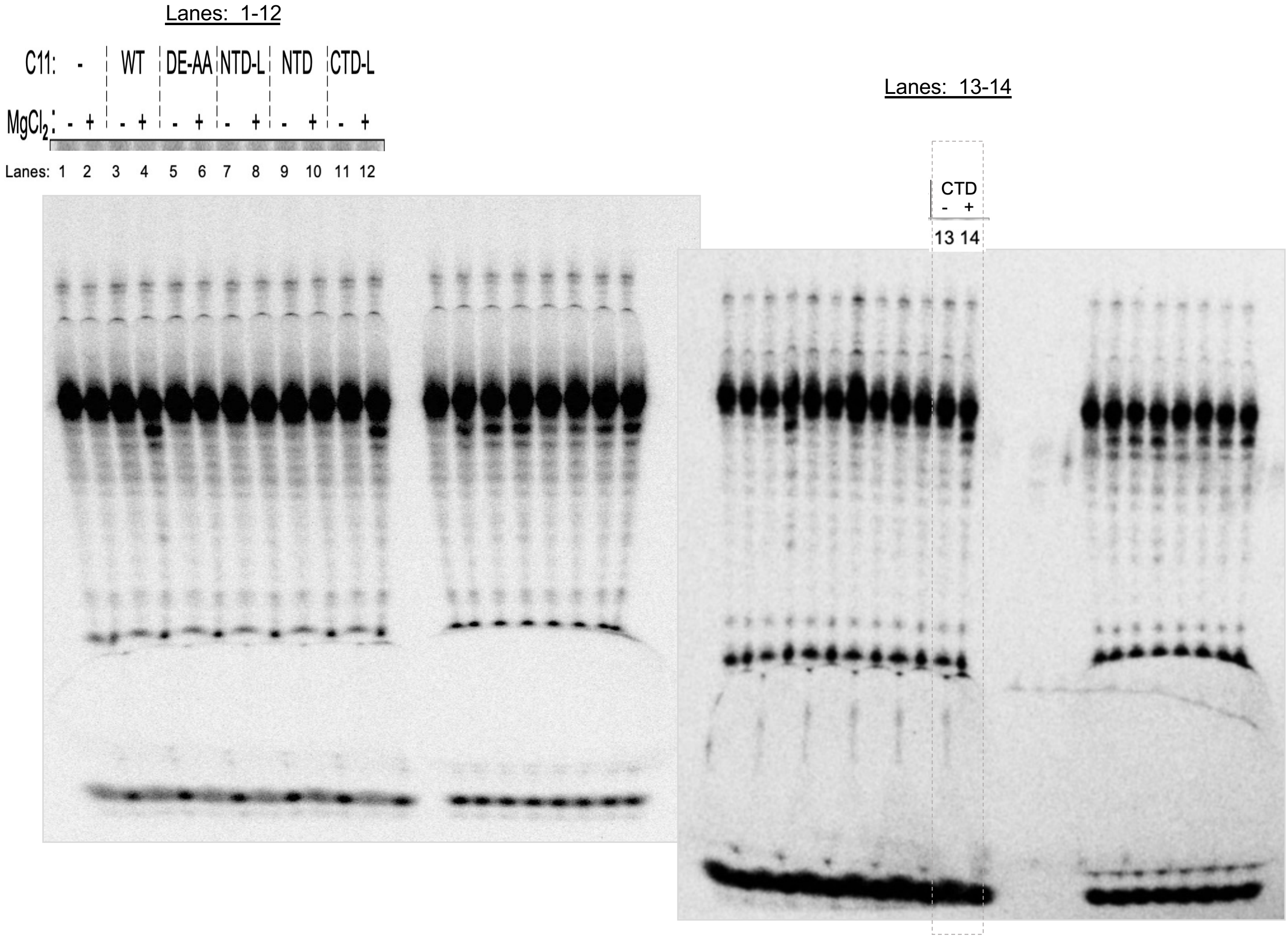

Uncropped gel for Fig 4B

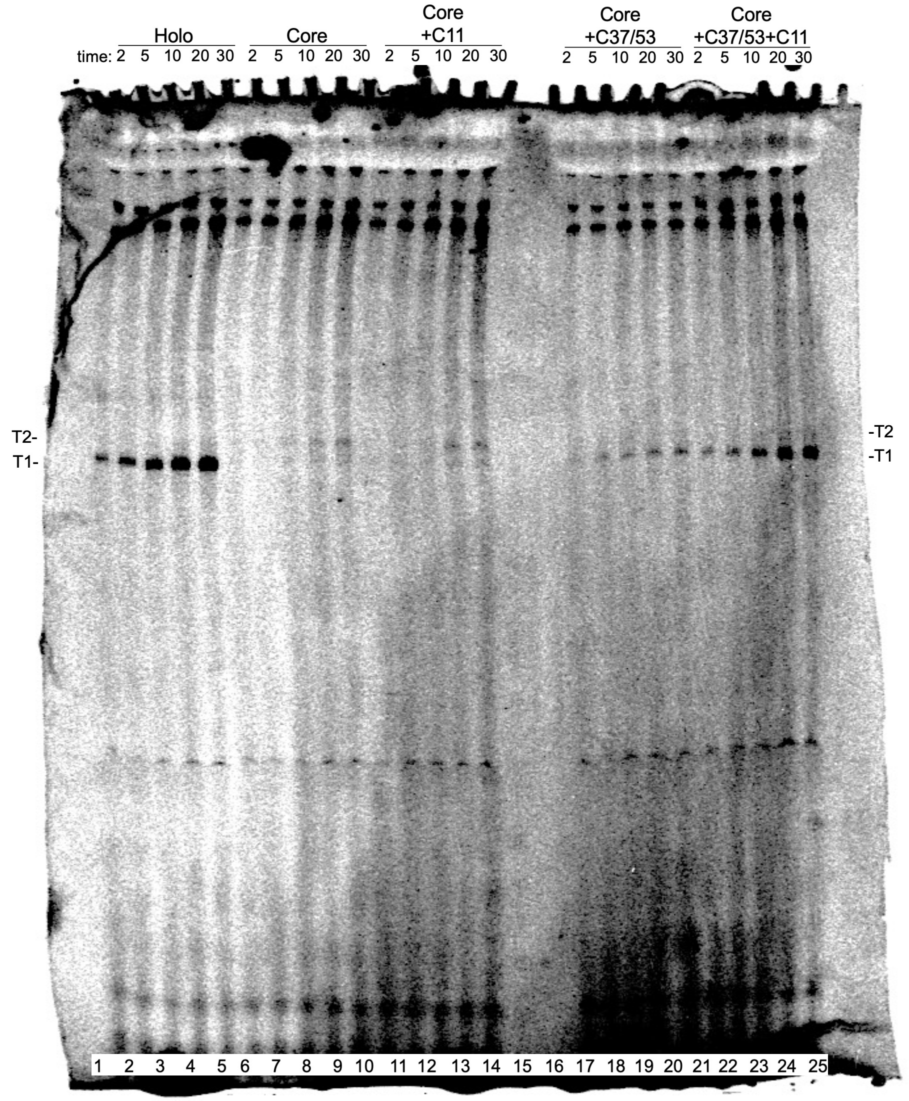

Uncropped gel for Fig 4D

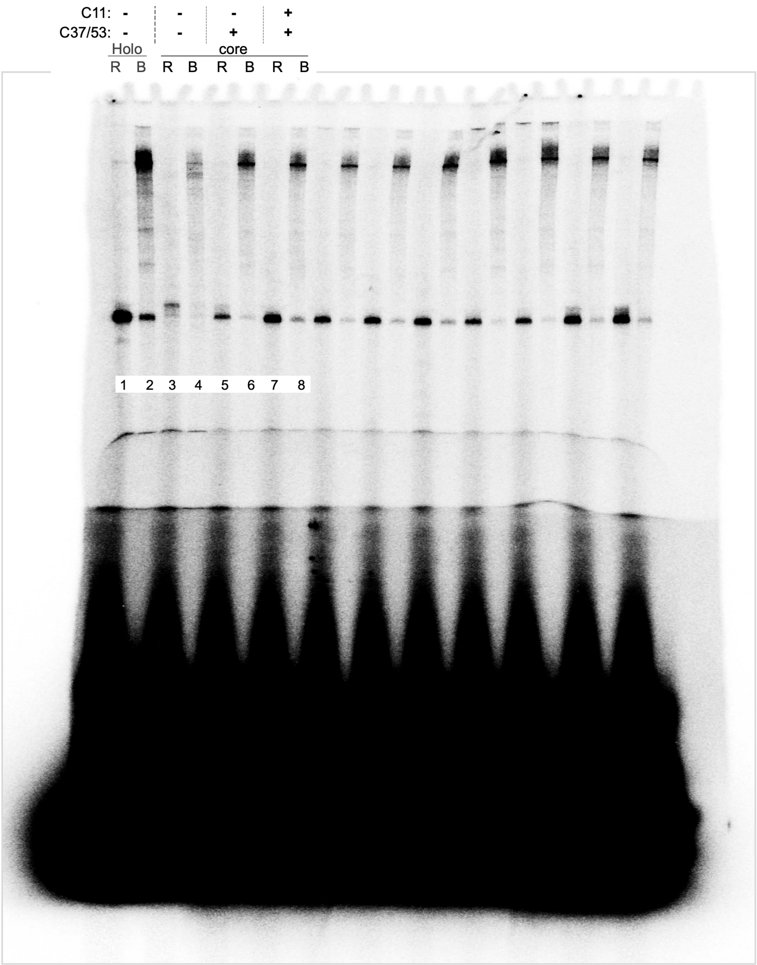

Uncropped gels for Fig 5A

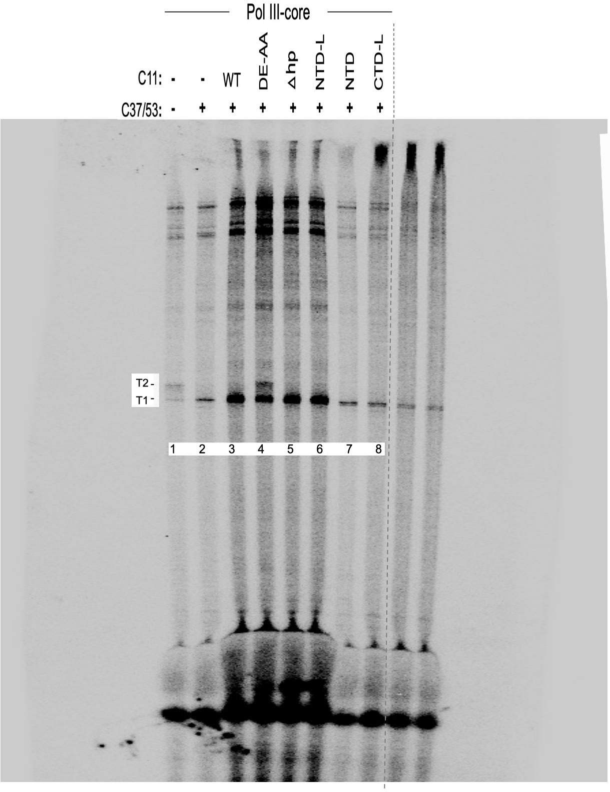

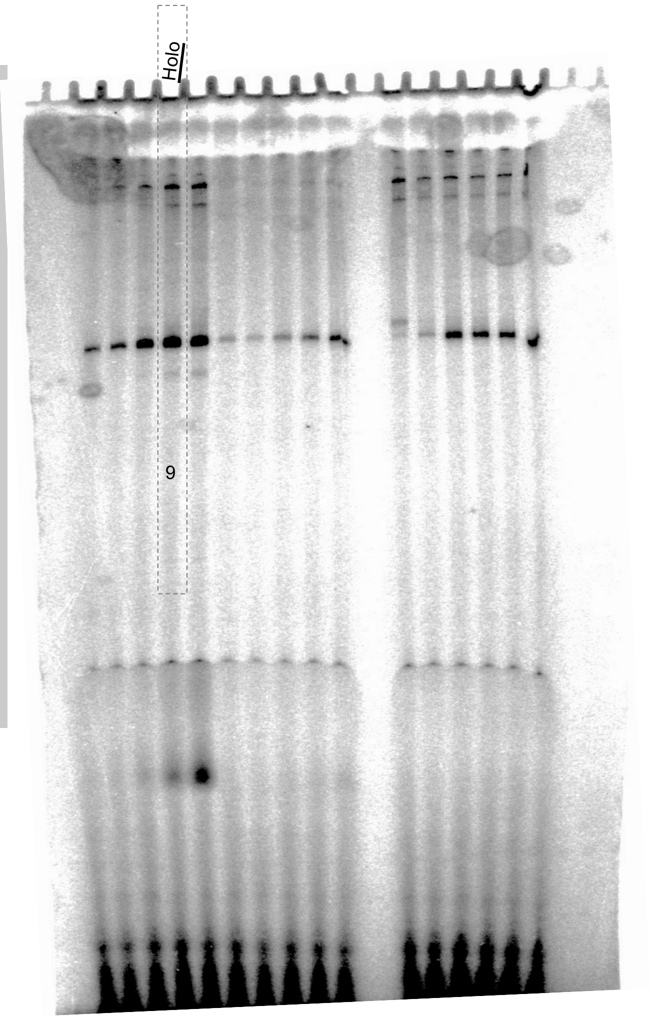

Uncropped gel for Fig 5C

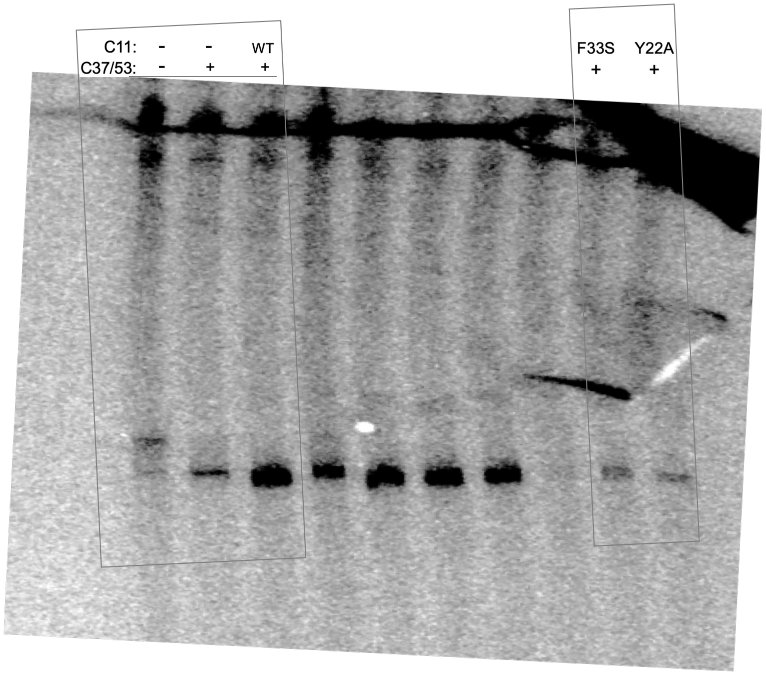

Uncropped gel for Fig 5D

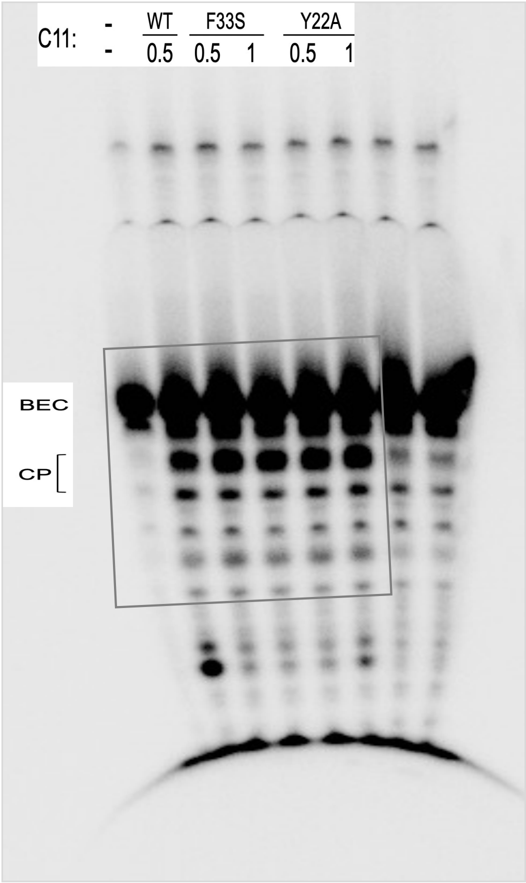

Uncropped gel for Fig 6A

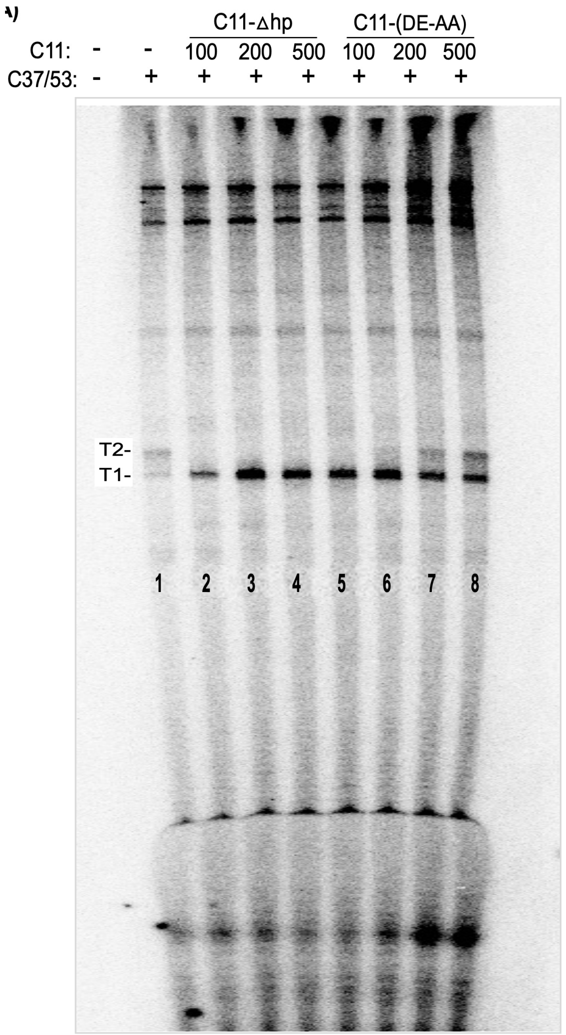

Uncropped images for Fig 6B

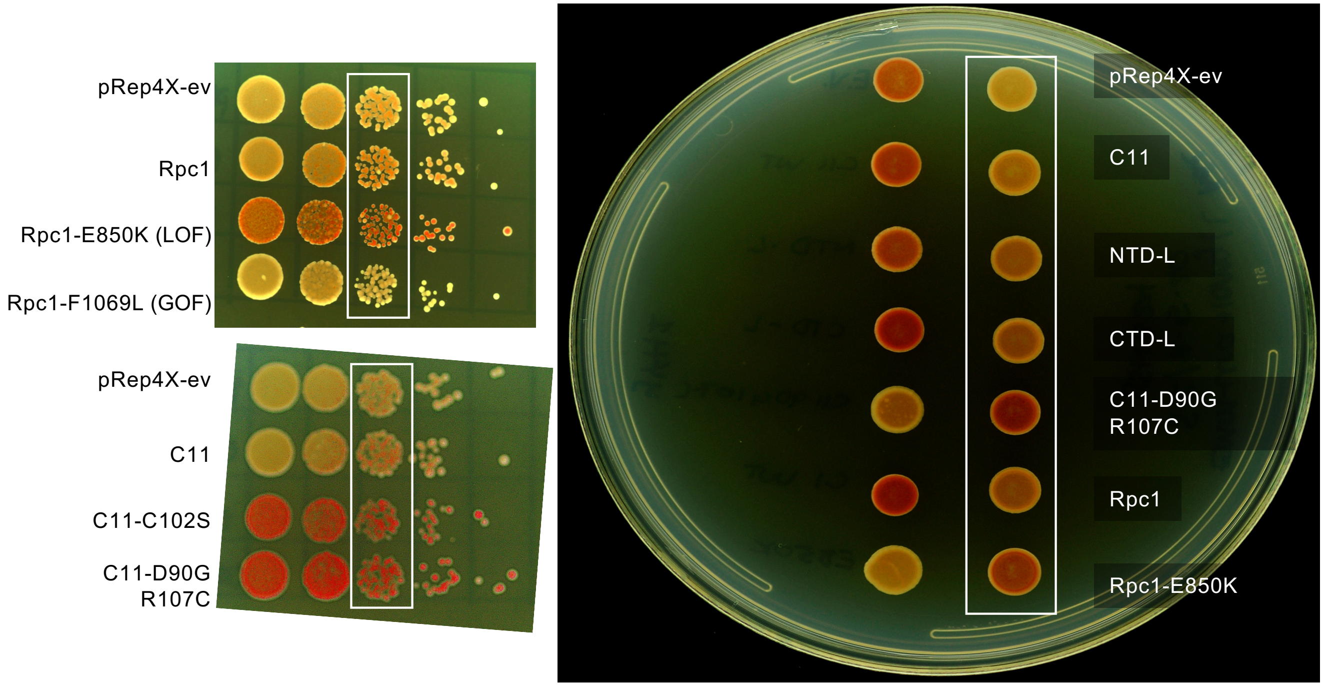

Uncropped gel for Fig 7A

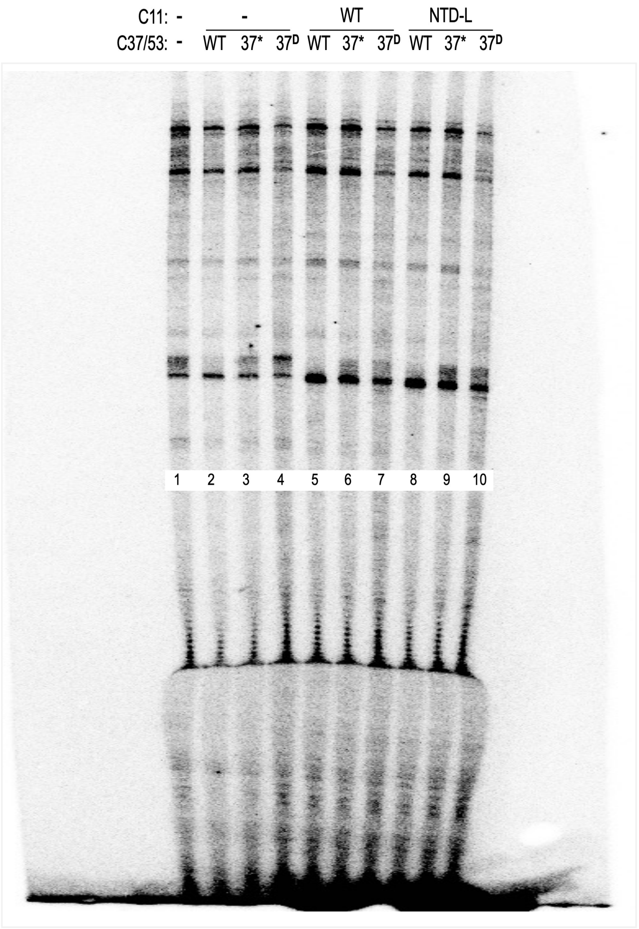

Uncropped gel for Fig 7B

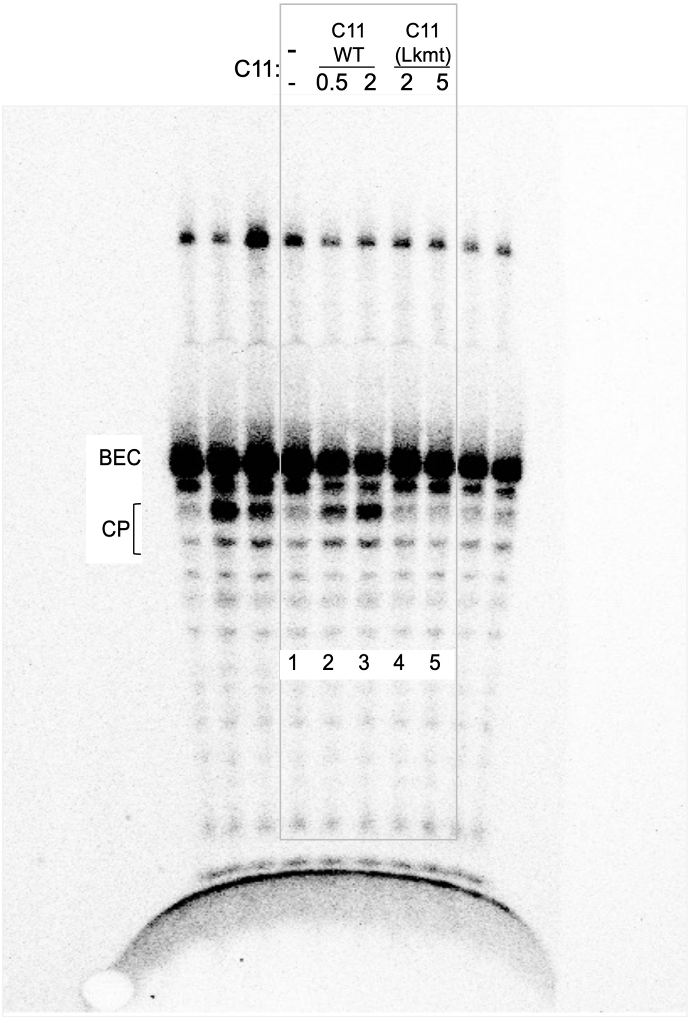

Uncropped gel image for Fig 7C

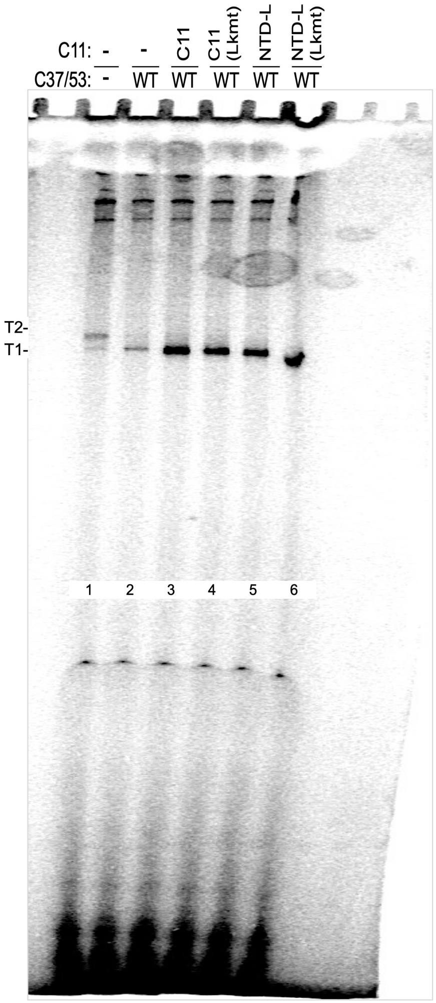

Uncropped images for Fig 8D

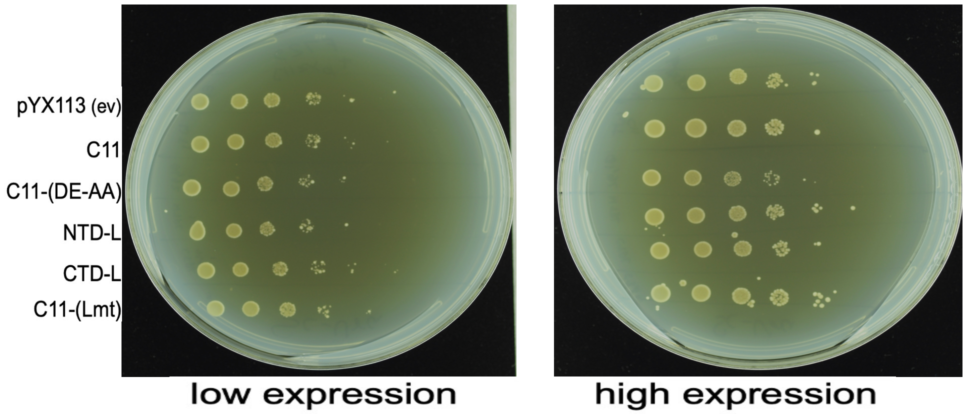

Uncropped blot for Fig 8E

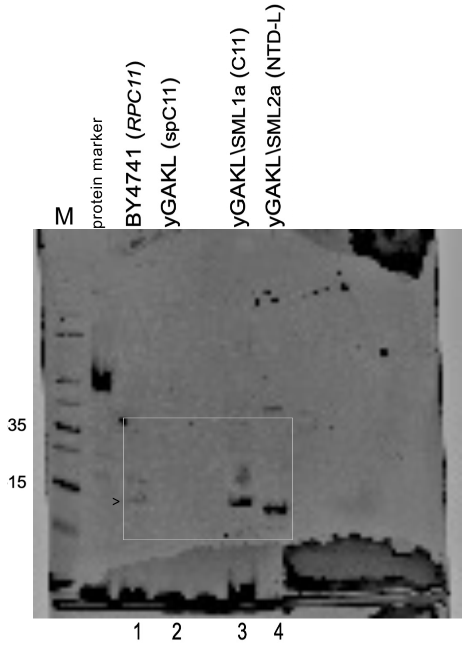

Uncropped blots for Fig 8F

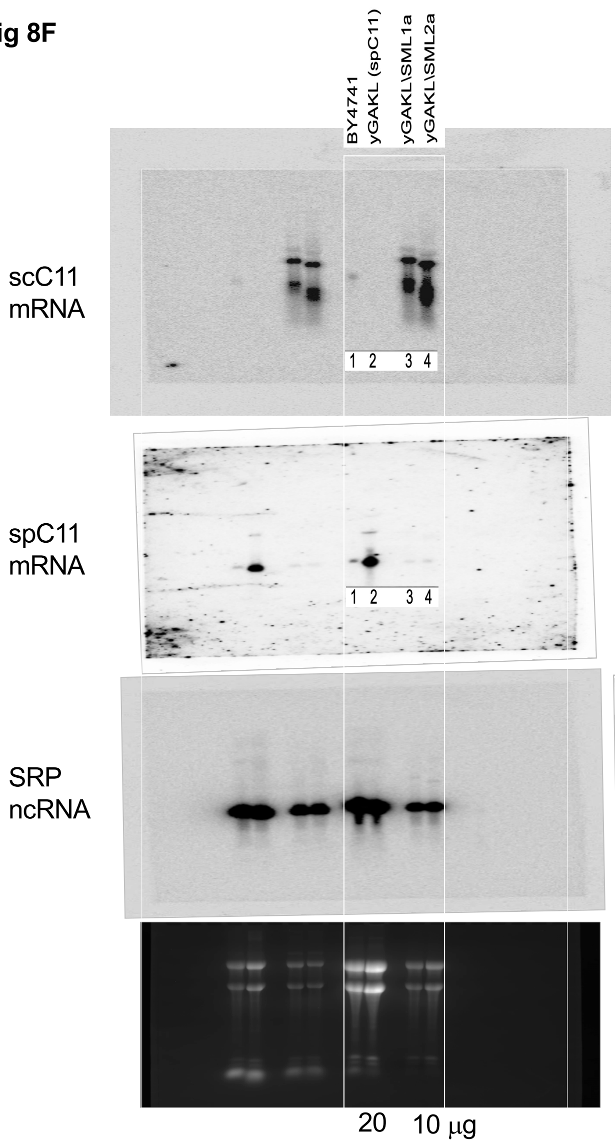

spC11 mRNA which comigrates with SRP ncRNA was probed after SRP ncRNA, the latter of which was not completely stripped

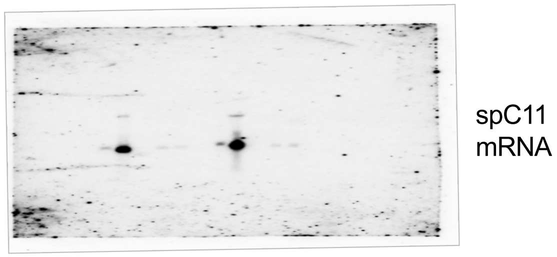

Uncropped gel for SUPPLEMENTARY Fig 4

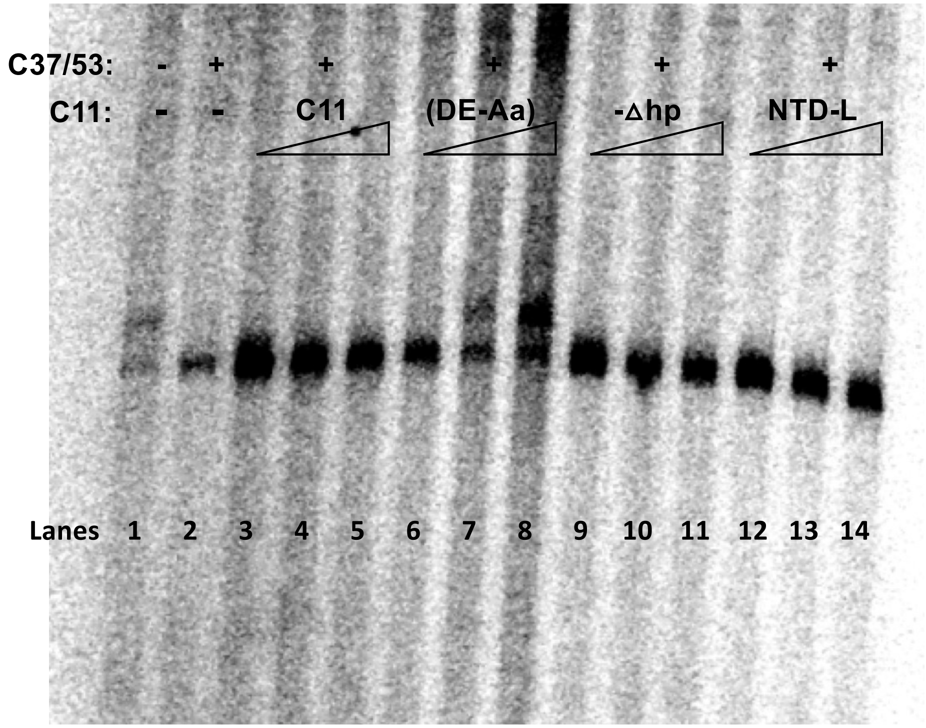

Uncropped gel for SUPPLEMENTARY Fig 2b

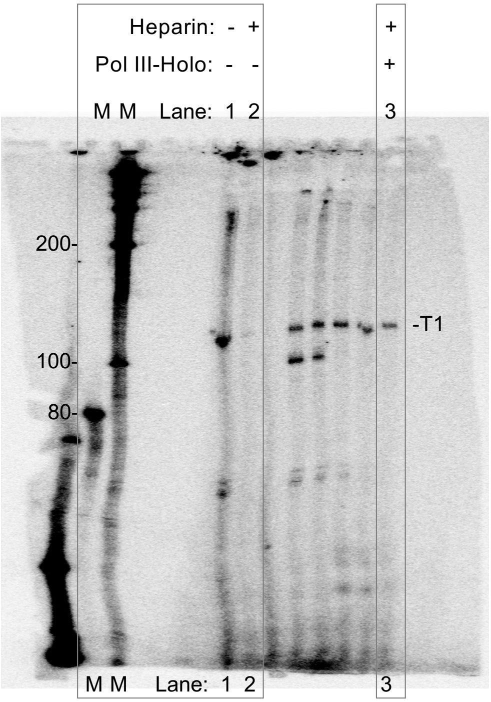

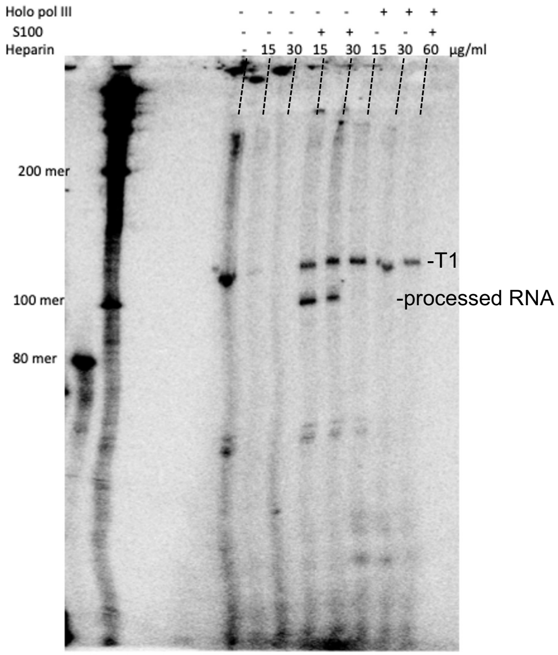

Uncropped gel for SUPPLEMENTARY Fig 2c

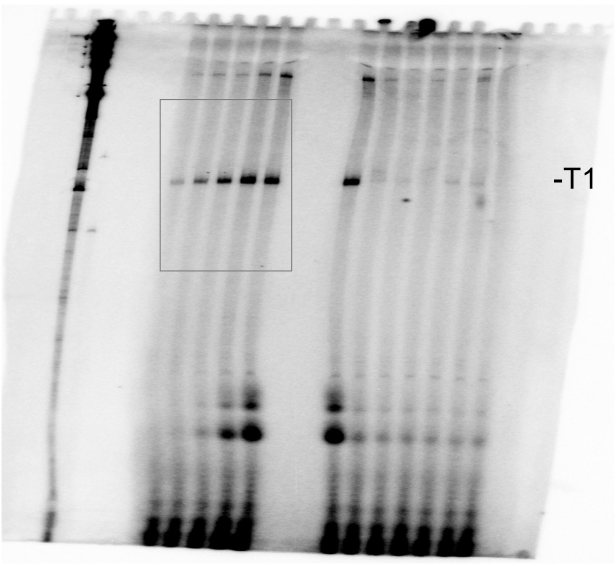

Uncropped gel for SUPPLEMENTARY Fig 1a

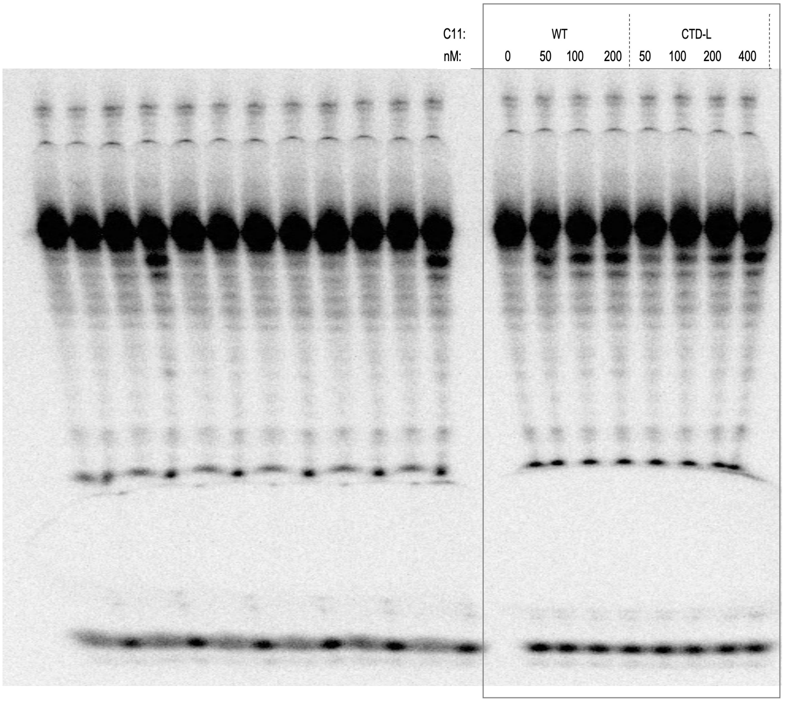

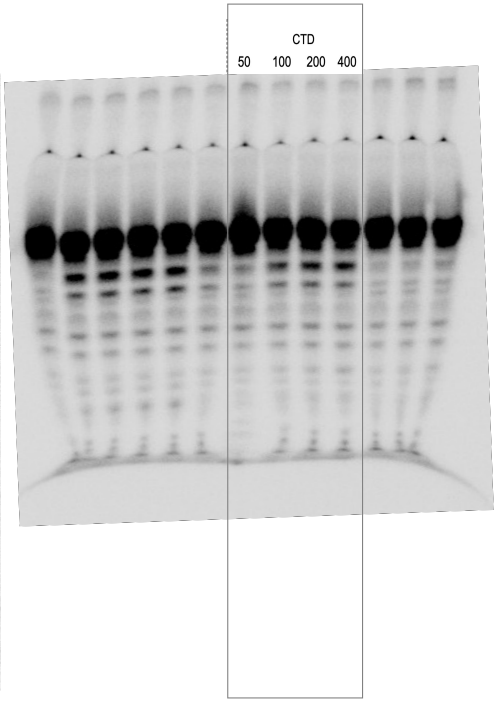

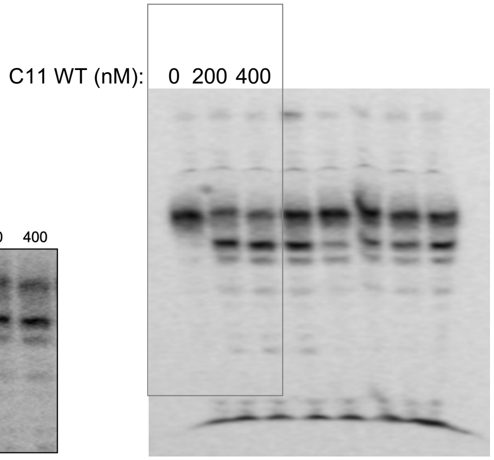

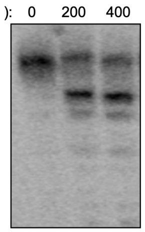

Concentation	WT C11		Avg	sdv	se
0	0	0	0	0	0
50	5	8	6.5	2.12132	1.500227
100	10	12	11	1.414214	1.000151
200	21	19	20	1.414214	1.000151
400	50	47	48.5	2.12132	1.500227

Concentation	WT CTD		AVG	SDV	
0	0	0	0	0	0
50	0	2	1	1.414214	1.0002
100	7	9	8	1.414214	1.0003
200	11	12	11.5	0.707107	0.5001
400	26	22	24	2.828427	2.0002

related to figure 2e

Concentation	WT CTDL				
0	0	0	0	0	0
50	0	0	0	0	0
100	5	7	6	1.414214	1.000151
200	12	14	13	1.414214	1.000151
400	21	25	23	2.828427	2.000302

% cleavage product (CP) =
CP/(BEC + CP) x 100

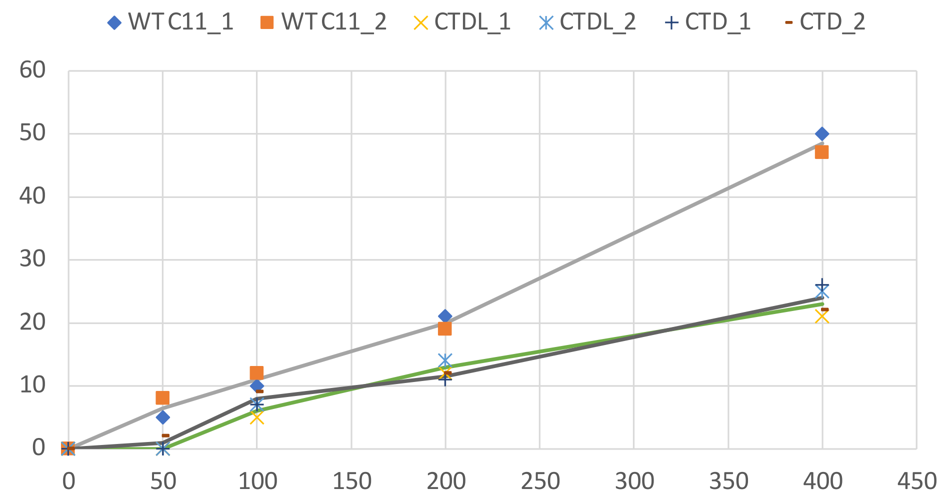

related to figure 3c

	1st exp	2nd	Avg	SDV	SE
Holo	59	47	53	8.485281	6.000906
Core	10	6	8	2.828427	2.000302
Core+C37/53	23	17	20	4.242641	3.000453
Core+C11	9	11	10	1.414214	1.000151
Core+M	10	12	11	1.414214	1.000151
Core+NTDL	8	12	10	2.828427	2.000302
Core+NTD	8	10	9	1.414214	1.000151
Core+CTDL	10	8	9	1.414214	1.000151
Core+C11+37	38	34	36	2.828427	2.000302
Core+M+37	37	33	35	2.828427	2.000302
Core+NTDL+37	38	34	36	2.828427	2.000302
Core+NTD+37	35	33	34	1.414214	1.000151
Core+CTDL+37	20.5	17.5	19	2.12132	1.500227
P value holo and core= .056					
P value CTDL and wtC11+37= .023					
P value for CTDL and NTDwith WT=.021					
p value for NTD and NTDL with WT = 0.29					
p value for core and core+c37/53 = 0.052929					
column H, line 5					

c)

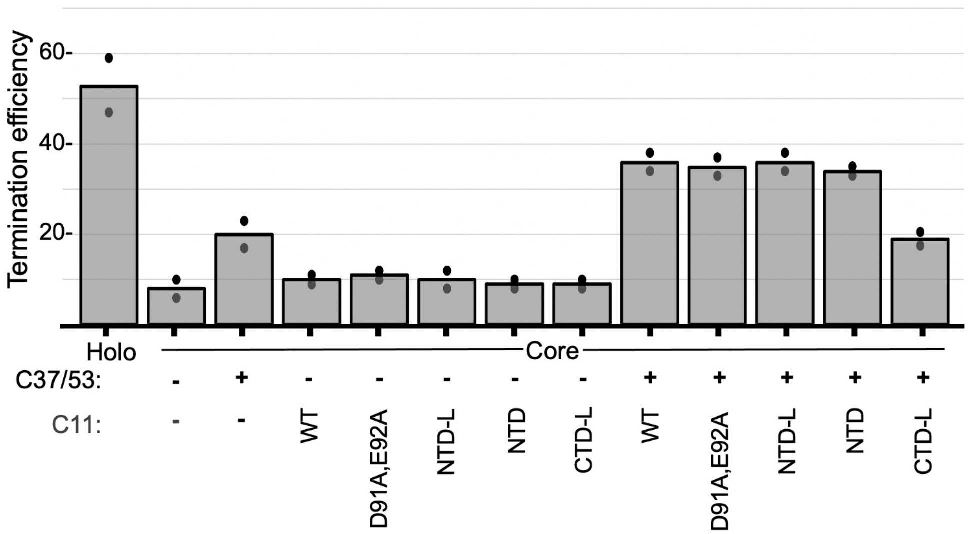

related to figure 4c

1		54511.1	1161	-------		-------		7.8	46.95	-------	-------	
2		63631.23	1161	-------		-------		9.11	54.81	-------	-------	
3		75085.98	1161	-------		-------		10.75	64.67	-------	-------	
4		94165.05	1161	-------		-------		13.48	81.11	-------	-------	
5		101771.9	1161	-------		-------		14.57	87.66	-------	-------	
6		47655.71	1161	-------		-------		6.82	41.05	-------	-------	
7		53542.4	1161	-------		-------		7.66	46.12	-------	-------	
8		61053.37	1161	-------		-------		8.74	52.59	-------	-------	
9		74697.09	1161	-------		-------		10.69	64.34	-------	-------	
10		72517.38	1161	-------		-------		10.38	62.46	-------	-------	
Image quant												
Time	Holo	Core	Core+C11	Core+C37/53	Core+C37/53/C11		Time	Holo	Core	Core+C11	Core+C37/53	Core+C37/53/C11
2	82325.6	4124.6	4956.3	5898.4	49228.1		2	80825.05	4140.65	4788.15	5838.2	49263.6
5	199275	8553.9	8877.5	20278.5	121345.2		5	204273	8403	8710.15	19828.5	121322.7
10	291402	131952.9	146563.3	144988.2	200595.9		10	296251.3	132447	146062.7	141993.7	195593.4
20	391012	139890.6	146069.9	159878.8	262303.2		20	390511.5	139375.8	146073	164839.8	262606.2
30	429190	141756.1	149888.5	170089.1	268839.9		30	424184.8	142253.6	149539	170054.1	263889.9
Time	Holo	Core	Core+C11	Core+C37/53	Core+C37/53/C11							
2	79324.5	4156.7	4620	5778	49299.1							
5	209271	8252.1	8542.8	19378.5	121300.2							
10	301101	132941	145562	138999.2	190590.9							
20	390011	138861	146076	169800.8	262909.2							
30	419980	142751	149888.5	170019.1	258939.9							

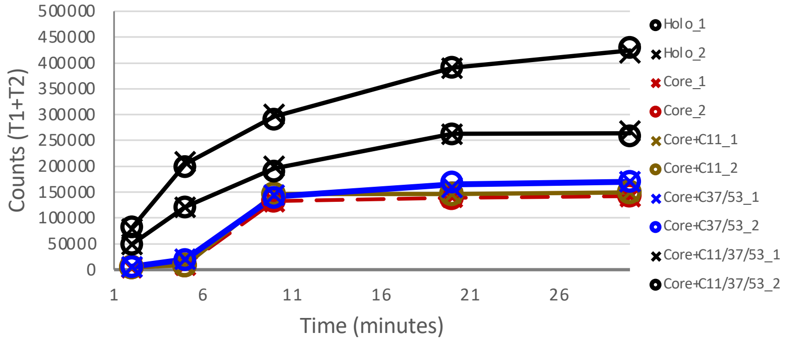

related to figure 6d

EV	1.1699	7.03E-03		EV	627300	631900		1.17692308	1.16286345		1.16989326	0.00994166	0.00703087		533000	543400
C11	1.0951	0.0432		C11	577000	546500		1.13829158	1.05197305		1.09513231	0.06103641	0.04316578		506900	519500
C11-D90GR107C	0.6631	4.83E-03		90,107	486700	522900		0.65823641	0.66790139		0.6630689	0.00683418	0.00483322		739400	782900
C11-C102S	0.7229	0.044		C102S	404100	431000		0.67893145	0.76690391		0.72291768	0.06220593	0.04399287		595200	562000
C11-NTDL	1.286	0.0394		NTDL	1055000	1066000		1.24660286	1.3253761		1.28598948	0.05570109	0.03939257		846300	804300
C11-CTDL	1.1192	0.0261		CTDL	569000	557400		1.14533011	1.09315552		1.11924282	0.03689301	0.02609124		496800	509900
C1	1.0677	0.0313		C1	937700	994600		1.03636163	1.09900552		1.06768358	0.04429593	0.03132668		904800	905000
C1-E850K	0.6214	0.0801		E850K	354400	331800		0.70150435	0.54127243		0.62138839	0.11330108	0.08012806		505200	613000

Time in hrs	C11 WT	C11WT	Avg	SDV	SE
0	0.05	0.05	0.05	0	0
4	0.065	0.07	0.0675	0.0035355	0.0025004
8	0.09	0.094	0.092	0.0028284	0.0020003
12	0.19	0.18	0.185	0.0070711	0.0050008
18	0.476	0.431	0.4535	0.0318198	0.0225034
24	0.85	0.79	0.82	0.0424264	0.0300045
30	1.41	1.42	1.415	0.0070711	0.0050008
36	1.98	1.86	1.92	0.0848528	0.0600091
42	2.1	2.01	2.055	0.0636396	0.0450068
48	2.12	2.09	2.105	0.0212132	0.0150023

Time in hrs	C11(52,53)				
0	0.05	0.05	0.05	0	0
4	0.052	0.051	0.0515	0.0007071	0.0005001
8	0.07	0.065	0.0675	0.0035355	0.0025004
12	0.09	0.075	0.0825	0.0106066	0.0075011
18	0.226	0.205	0.2155	0.0148492	0.0105016
24	0.481	0.401	0.441	0.0565685	0.040006
30	0.85	0.81	0.83	0.0282843	0.020003
36	1.31	1.41	1.36	0.0707107	0.0500076
42	1.5	1.51	1.505	0.0070711	0.0050008
48	1.55	1.58	1.565	0.0212132	0.0150023

Time in hrs	NTDL				
0	0.05	0.05	0.05	0	0
4	0.051	0.052	0.0515	0.0007071	0.0005001
8	0.061	0.061	0.061	0	0
12	0.071	0.07	0.0705	0.0007071	0.0005001
18	0.17	0.19	0.18	0.0141421	0.0100015
24	0.28	0.35	0.315	0.0494975	0.0350053
30	0.45	0.49	0.47	0.0282843	0.020003
36	0.75	0.65	0.7	0.0707107	0.0500076
42	1.2	1.05	1.125	0.106066	0.0750113
48	1.23	1.09	1.16	0.0989949	0.0700106

Time in hrs	(NTDL52,53)				
0	0.05	0.05	0.05	0	0
4	0.05	0.052	0.051	0.0014142	0.0010002
8	0.06	0.06	0.06	0	0
12	0.071	0.065	0.068	0.0042426	0.0030005
18	0.158	0.12	0.139	0.0268701	0.0190029
24	0.25	0.231	0.2405	0.013435	0.0095014
30	0.44	0.41	0.425	0.0212132	0.0150023
36	0.761	0.661	0.711	0.0707107	0.0500076
42	1.11	1.04	1.075	0.0494975	0.0350053
48	1.2	1.05	1.125	0.106066	0.0750113

related to figure 8c
